# Identification of Variants of Hepatitis C Virus (HCV) Entry Factors in Patients Highly Exposed to HCV but Remaining Uninfected: An ANRS Case-Control Study

**DOI:** 10.1371/journal.pone.0142698

**Published:** 2015-11-16

**Authors:** Baptiste Fouquet, Jade Ghosn, Yann Quertainmont, Dominique Salmon, Christophe Rioux, Claudine Duvivier, Jean-François Delfraissy, Micheline Misrahi

**Affiliations:** 1 Univ Paris Sud, Faculté de Médecine, Hôpitaux Universitaires Paris Sud, Hopital Bicetre, 94275, Le Kremlin-Bicêtre, France; 2 APHP, Unité Fonctionnelle de Thérapeutique en Immuno-Infectiologie, Hôpitaux Universitaire Paris Centre, Hôpital Hôtel Dieu, Paris, France; 3 Université Paris Descartes, EA 7327, Faculté de Médecine site Necker, Sorbonne Paris Cité, Paris, France; 4 APHP, Service de Médecine Interne, Hôpitaux Universitaires Paris Sud, Hôpital Bicêtre, 94275, Le Kremlin-Bicêtre, France; 5 APHP, Service de Médecine Interne, Hôpitaux Universitaires Paris Centre, Hôpital Cochin, Paris, France; 6 APHP, Service des Maladies Infectieuses et Tropicales, Hôpitaux Universitaires Paris Nord val de Seine, Hospitalier Bichat-Claude Bernard, Paris, France; 7 Centre Médical de l’Institut Pasteur, 75015, Paris, France; SRI International, UNITED STATES

## Abstract

Hepatitis C virus (HCV) causes persistent infection in 75% of cases and is a major public health problem worldwide. More than 92% of intravenous drug users (IDU) infected by human immunodeficiency virus type 1 (HIV-1) are seropositive for HCV, and it is conceivable that some HIV-1-infected IDU who remain uninfected by HCV may be genetically resistant.Here we conducted a case-control study to identify mutations in HCV entry coreceptors in HIV-infected IDU who remained uninfected by HCV. We recruited 138 patients, comprising 22 HIV+ HCV- case IDU and 116 HIV+ HCV+ control IDU. We focused on coreceptors in which point mutations are known to abolish HCV infectivity *in vitro*. Our previous study of the Claudin-1 gene revealed no specific variants in the same case population. Here we performed direct genomic sequencing of the Claudin-6, Claudin-9, Occludin and Scavenger receptor-B1 (SCARB1) gene coding regions. Most HIV+ HCV- IDU had no mutations in HCV coreceptors. However, two HIV+ HCV- patients harbored a total of four specific mutations/variants of HCV entry factors that were not found in the HIV+ HCV+ controls. One case patient harbored heterozygous variants of both Claudin-6 and Occludin, and the other case patient harbored two heterozygous variants of SCARB1. This suggests that HCV resistance might involve complex genetic events and factors other than coreceptors, a situation similar to that reported for HIV-1 resistance.

## Introduction

Hepatitis C virus (HCV) infects more than 170 million individuals worldwide and is a major public health problem. HCV infection becomes persistent in 75% of cases. Chronic carriers frequently develop fibrosis, cirrhosis and, in some cases, hepatocellular carcinoma, especially if untreated. Because human immunodeficiency virus (HIV) and HCV share common routes of infection, coinfection with HCV is frequent among HIV-1-infected patients: in Europe, about one-third of all HIV-1-infected individuals have anti-HCV antibodies [1, 2]. However, the seroprevalence of HCV among HIV-1-infected individuals in France ranges from 8% when HIV-1 is transmitted via heterosexual contact to 41.7% among hemophiliacs and/or transfusion recipients and more than 92% among intravenous drug users (IDU) [3]. Thus, individuals who acquired HIV-1 via intravenous drug use but remain uninfected by HCV are rare.

HCV entry into hepatocytes is complex and not fully understood [4, 5]. It is a multistep process involving several host factors and coreceptors, including heparan sulfate [6], the tetraspanin CD81 [7, 8], the scavenger receptor B1 (SCARB1) [9], the tight-junction proteins claudin-1 (CLDN1) [10] 6 and 9 (CLDN6 and CLDN9) [11] and occludin (OCLN) [12]. The EGF and EphrinA2 receptors, the Niemann-Pick C1-like 1 cholesterol absorption receptor [13, 14] and, more recently, transferrin receptor 1 (TfR1) and cell-death-inducing DFFA like effector B (CIDEB) have all been reported to play a role in HCV entry [5]. Infectious virions complete their binding, endocytosis and fusion processes through sequential interactions with SR-B1 and CD81 earlier in the entry pathway, whereas the two tight junction proteins (claudin-1 and occludin) play important roles during a post-binding step [15]. Claudin-1 has also been shown to play a key role in cell-to-cell virus transmission [16]: no evidence of claudin-1-independent HCV entry has so far been reported [10, 17].

The aim of this case-control study was to identify mutations in HCV entry coreceptors in long-term HIV-infected intravenous drug users who remained uninfected by HCV. As previously described [18], we recruited 138 patients having acquired HIV-1 infection before 1995 through intravenous drug use. Cases (n = 22) were HIV-1-infected IDU who remained negative for anti-HCV antibodies and HCV RNA despite at least five years of intravenous drug use. Controls (n = 116) were HIV-1-infected IDU coinfected with HCV (anti-HCV antibody-positive).

We focused on coreceptors in which point mutations are known to prevent HCV infection *in vitro* [19–22]. In a previous case-control study of the CLDN1 gene in the same HIV-infected IDU population, we identified no specific coding variants in the HCV-uninfected case population [18]. Here we focused on the CLDN6, CLDN9, OCLN and SCARB1 coding regions.

## Materials and Methods

### Study participants

We conducted a case-control study of HIV-infected individuals followed in four Parisian clinical centres [18]. The objective was to compare the sequences of HCV entry factor genes between HIV-1-infected, HCV-uninfected IDU and HIV/HCV-coinfected IDU. Cases were defined as: (i) having acquired HIV-1 infection before 1995 through intravenous drug use, (ii) having injected illicit drugs for at least 5 years, and (iii) being negative for anti-HCV antibodies and HCV-RNA. Controls were defined as: (i) having acquired HIV-1 infection before 1995 through intravenous drug use, (ii) having injected illicit drugs for at least 5 years, and (iii) being positive for anti-HCV antibodies and HCV-RNA before any anti-HCV treatment. The case-control ratio was 1:5.

Patients 18 years of age or older were eligible for participation in this study if they had acquired HIV-1 through intravenous drug use before 1995, i.e. before substitution programs with methadone and buprenorphine were launched in France. Data on the HIV-1 transmission group and date of HIV-1 diagnosis were collected from the databases of the four clinical centres. The clinical charts of potentially eligible patients were then reviewed by the same investigator to check for injection drug history (including the date of first i.v. drug use). The study was restricted to individuals who did not have HIV-1-seropositive sexual partners at the time of HIV diagnosis and who were not transfusion recipients.

The Scientific Review Board of Bicêtre Hospital approved the study and all the patients gave their written informed consent to participate.

### Sequencing of the CLDN6, CLDN9, OCLN and SCARB1 genes

**Fig 1:**
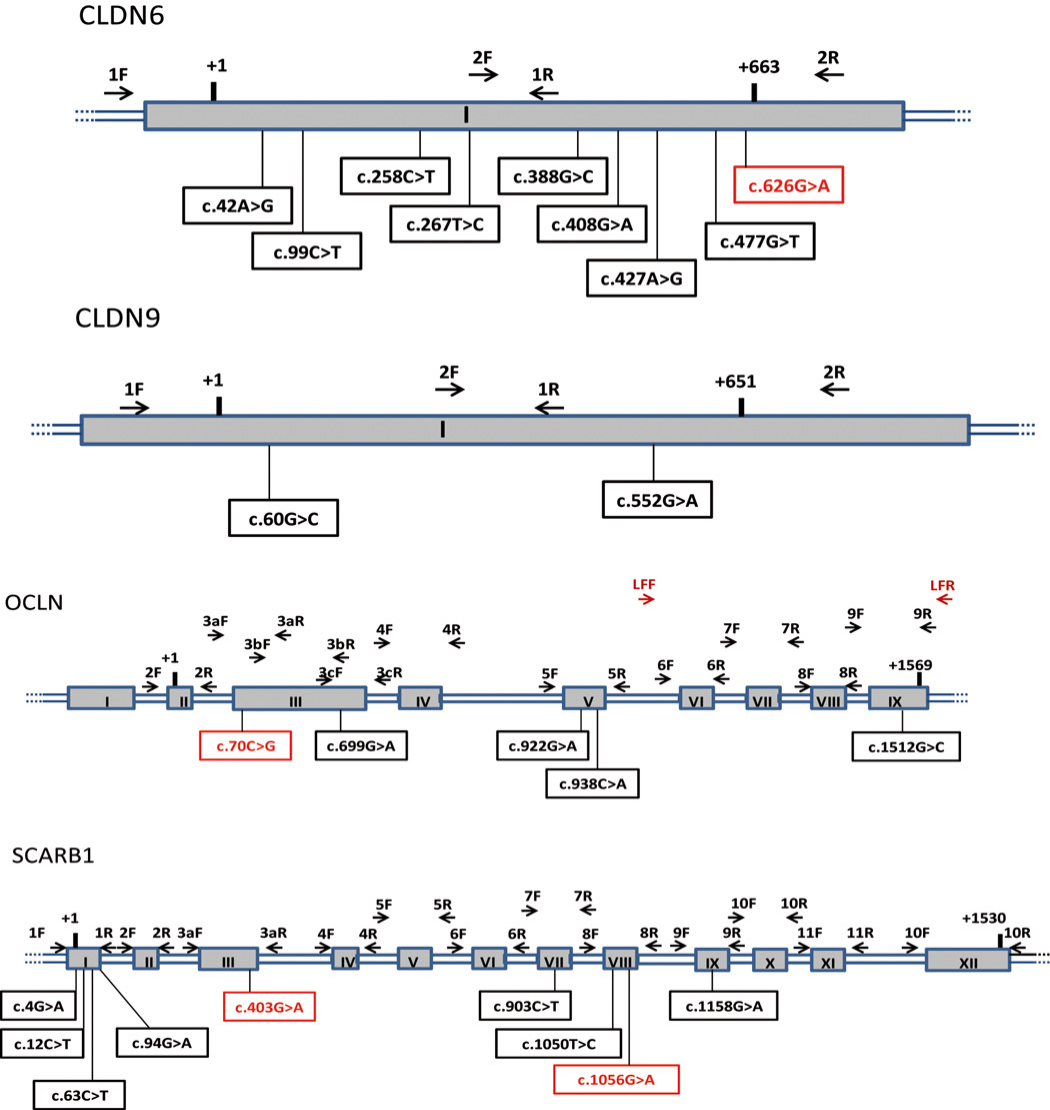
Schematic representation of of CLDN6, CLDN9, OCLN and SCARB1 genes. Exons are represented by numbered gray rectangles and introns or non coding regions by double blue lines. Positions of primers pairs used for direct genomic sequencing are shown. Reference sequences of the transcripts are NM_021195.4 (CLDN6), NM_020982.3 (CLDN9), NM_002538.3 (OCLN) and NM_005505.4 (SCARB1). The numbering starts at the first base of the initiation codon ATG, and stops at the first base of the termination codon of the corresponding transcripts. The sequences of corresponding primers are shown in Table 1 and Table 2. F: Forward primer, R: Reverse primer, LR: Long Range PCR primer. SNPs identified by direct genomic sequencing are indicated, in red SNPs specific of the case population HIV+ HCV-.

DNA was extracted from peripheral blood mononuclear cells with the Blood and Cell Culture DNA maxi kit (Qiagen). Forward (F) and reverse (R) primer pairs were designed to amplify all the exons and intron-exon junctions of each gene (Table 1, Table 2 and Fig 1). Each exon was amplified with the corresponding F/R primer pair in classical polymerase chain reaction (PCR) conditions: 5 min at 94°C followed by 30 cycles of 30 s at 95°C, the hybridization temperature being adjusted to each exon (Table 1 and Table 2), followed by a final elongation step of 8 min at 72°C.

**Table 1:** Primers used to sequence the CLDN6, CLDN9 and OCLN genes.

Primer	exon	Sequence 5'-3'	Hybridation T°C	amplicon size bp
** **	** **	**CLDN6 gene**	** **	** **
**1F**	1	CTTGTTGTGCTTCTGTCCCA	56	439
**1R**	1	AGGACCCCTGAGATGACAAA	56	
**2F**	1	CTTGCTGGTCTACCTTGCTG	56	467
**2R**	1	AAAAGGTACGAACCCATCCC	56	
** **	** **	**CLDN9 gene**	** **	** **
**1F**	2	CACACCAGACACACCCTCTG	58	478
**1R**	2	AGCACACAGGGATGAGCAC	58	
**2F**	2	GTGTACCACGTGTGTGGAGG	58	440
**2R**	2	AGGAGGTTGTGATGGAGCAG	58	
** **	** **	**OCLN gene**	** **	** **
**2F**	2	GCAGTGAGCTGTGATTGGA	59	340
**2R**	2	GCAAACACTTAAAGTTTCAACC	59	
**3Fa**	3	CCAAATAAGTTGTGTTCTTTCTGC	54	356
**3Ra**	3	CCAAAGCCACTTCCTCCATA	54	
**3Fb**	3	TCTCCTCCAGGAGTGATTCG	56	373
**3Rb**	3	ATGCCCAGGATAGCACTCAC	56	
**3Fc**	3	CGCGTTGGTGATCTTTGTTA	56	328
**3Rc**	3	TTGAAGGCCTCTGCTAAGGA	56	
**4F**	4	CATTAGGCATTTTCTGAGGATTG	60	586
**4R**	4	AACCATTTCCACTTAGCCCATC	60	
**5F**	5	TGTGGGCGTGAGATAATGAGACCA	56	595
**5R**	5	CCAGCTTTTGTGTGCACTGCTGG	56	
**LRF**	6–9	GGTTTGGTGAAGCATTTGCCTGTGAAG	68	23153
**LRR**	6–9	AACGACTAACCAGCACAGCATCCAAAG	68	
**6F**	6	TGGTGTTTATTATGGCTGTGC	62	372
**6R**	6	CAACACCTGGTTGGTCTCCT	62	
**7F**	7	TCTCCATACCCAACCAGCTT	62	337
**7R**	7	AGGATGCTGTACCTCCACA	62	
**8F**	8	CCTTCAGACCTTCCTGCTGA	62	311
**8R**	8	GAAAAGCTCTTCCTCCAGATG	62	
**9F**	9	CAGGCACCTTGCGTATTTTAC	62	352
**9R**	9	GCTCACAGAGGTTTGGCTTC	62	

**Table 2:** Primers used to sequence the SCARB1 gene.

Primer	exon	Sequence 5'-3'	Hybridation T°C	amplicon size bp
** **		**SCARB1 gene**		** **
**1F**	1	ATGGCGGGGCTTGTCTTGGC	64	617
**1R**	1	CCTGGCCTCCCTCGTGCTCT	64	
**2F**	2	CCACCACCTCCTATCCCAAG	64	366
**2R**	2	CCCCATCCCGTCCACTCTGA	64	
**3F**	3	GTGTTGGGTGGGGGAGAGC	64	527
**3R**	3	GACAGCACAGGGCCGAAAGC	64	
**4F**	4	AGAGGGTGGTTCTGGTGTCC	64	521
**4R**	4	AAGCCGGTTTGAGTCAGGTTC	64	
**5F**	5	CTCAGCCCAGAATGTTCAGAC	64	381
**5R**	5	CACTAACCCCACCTGCCCC	64	
**6F**	6	AGCCTGCCCTCTTCCCAC	60	374
**6R**	6	GCTACTGAGTCAAATCCACGA	60	
**7F**	7	TGGGTGGGGAGGCAGAGTC	60	460
**7Rb**	7	GCCAGAGATTAAGCAGACAGC	60	
**8F**	8	TCCTGCCTCACCCCTTCTCT	60	440
**8R**	8	CTTCCCACCACCCCAGCC	60	
**9F**	9	GACGCCCACCCTCTTGACTG	60	240
**9R**	9	GGACCACTGGAGCACTGAGC	60	
**10F**	10	GGTGAGGGTTTAGTGTGTGC	60	327
**10R**	10	AGGGTGAAGTTTCTGATACGC	60	
**11F**	11	AGGCGGGCACAGAGGAAGG	60	449
**11R**	11	CAGGCAGAGTAGTGGCAACG	60	
**12F**	12	ATCGTTGAGGGTTGTTGGAC	60	362
**12R**	12	GCTGAAGGAATGAGCAGGAC	60	

To avoid amplification of the pseudogene for exons 6 to 9 of the OCLN gene, long-range (LR) PCR (Qiagen LongRange PCR Kit) was used as an intermediate step before PCR amplification of the exon. LR PCR amplification consisted of 10 min at 94°C followed by 30 cycles of 20 s at 94°C, 1 min at 68°C and 5 min at 72°C, and a final 10-min elongation step at 72°C. The amplicon was purified and used as a matrix to amplify *OCLN* exons 6 to 9, as described above.

Sequencing was performed on an ABI 3100 automated sequencer (Applied Biosystems, Foster City, CA, USA) according to the manufacturer’s instructions. Rare variants were confirmed by resequencing independent PCR products.

As a predicted splice site due to c.626G>A mutation in the CLDN6 gene (NM_021195.4) was detected, peripheral white blood cells were collected in a PAXgen Blood RNA collection tube (Qiagen) from the patient concerned, and RNA was extracted with a nucleic acid purification kit (PAXgen Blood RNA Kit, Qiagen). Then RT PCR was applied to the corresponding cDNAs using the RETROscript reverse transcription kit (Ambion, Lifes Technologies). PCR products were sequenced with primers corresponding to the C terminal part of *CLDN6*. All variants were verified by double-strand sequencing of two independent PCR products. The c.70C>G mutation in the OCLN gene (NM_002538.3) creates a new restriction site for BseX1. This mutation was confirmed by BseX1 (Fermentas, Thermo Scientific) restriction analysis of a PCR product.

### Bioinformatics analyses

Polyphen and SIFT (Sort Intolerant From Tolerant) are sequence homology based tools that predicts whether an amino acid substitution in a protein will have a phenotypic effect. These prediction softwares are based on the principle that protein evolution is correlated with protein function. Positions important for function should be conserved in an alignment of the protein family, whereas unimportant positions should appear diverse in an alignment. This software will predict which mutants may have a phenotypic effect.

SIFT takes a query sequence or a SNP and uses multiple alignment information to predict tolerated (meaning no predicted effect on structure or protein function) and deleterious (that can render the resulting protein nonfunctional or defective) substitutions for every position of the query sequence or for the SNP. From a query sequence SIFT searches for similar sequences, chooses closely related sequences that may share similar function to the query sequence, obtains the alignment of these chosen sequences, and calculates normalized probabilities for all possible substitutions from the alignment. Positions with normalized probabilities less than 0.05 are predicted to be deleterious, those greater than or equal to 0.05 are predicted to be tolerated (http://sift.jcvi.org/).

Polyphen-2 (http://genetics.bwh.harvard.edu/pph2/) predicts the functional significance of an allele replacement from its individual features using HumDIv and HumVar datasets (compiled human disease-causing mutations from the UniProtKB database). For a mutation, PolyPhen-2 calculates probability that this mutation is damaging and reports estimates of false positive rate (the chance that the mutation is classified as damaging when it is in fact non-damaging) and true positive rate (the chance that the mutation is classified as damaging when it is indeed damaging), mutation is also appraised qualitatively, as benign (correspond to tolerated for SIFT), possibly damaging, or probably damaging (damaging corresponds to deleterious for SIFT). Higher the score is higher the mutation is predicted damaging. The prediction confidence depends on rates of false and true positive and allows to qualify the mutation of possibly, probably or benign.

## Results

A total of 138 Caucasian patients were enrolled between February and December 2008, comprising 22 cases (20 men) and 116 controls (93 men). They were all followed in four Parisian clinical centers managing patients infected with HIV-1. Their median age was 46 years. The median year of first documented HIV-1 seropositivity was 1989 (range 1984–1994). They all reported using intravenous drug use for more than five years before 1995.

In the control patients (n = 116), the estimated median year of HCV acquisition (corresponding to the first year of intravenous drug use) was 1992 (range 1982–1994). The distribution of the main HCV genotypes was as follows: 60% genotype 1, 20% genotype 3, 10% genotype 4 and 7% genotype 2. CD4 T cell count did not differ between the case and the control populations at the time of first diagnosis of HIV infection.

In our previous study of the CLDN1 gene, we identified no specific coding variants in the case population [18]. Here, direct genomic sequencing of the whole coding region and adjacent intron-exon junctions of the CLDN6, CLDN9, OCLN and SCARB1 genes in the case and control populations enabled us to identify several new mutations and single nucleotide polymorphisms (SNPs).

**Fig 2:**
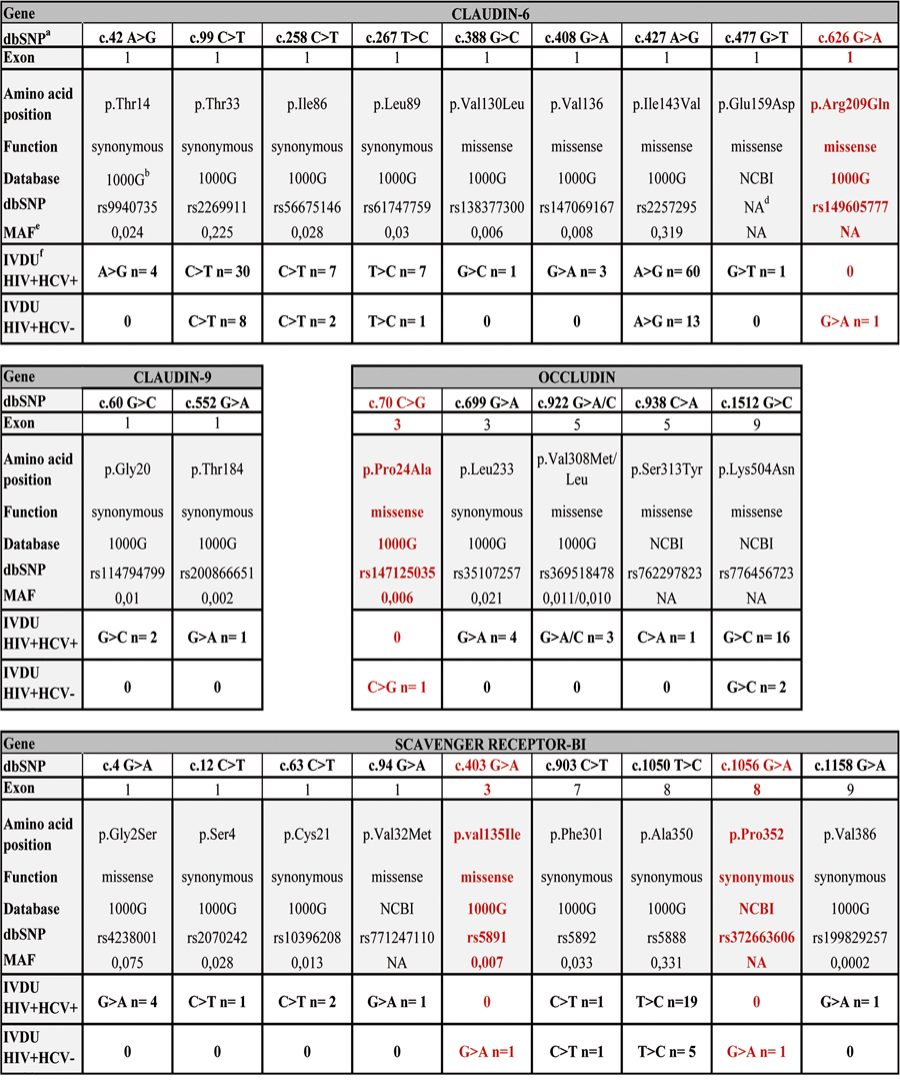
Variants of HCV entry factors found in HIV-infected, HCV-uninfected patients but not in HIV/HCV-coinfected controls. ^a^ Database single nucleotide polymorphism. ^b^ 1000 Genomes database. ^c^ refSNP reference identification number of single nucleotide polymorphism. ^d^ Not attributed. ^e^ Minor allele frequency. ^f^ Intravenous drug users.

In the CLDN6 gene, we found SNPs in both populations, corresponding to frequent polymorphisms described in conventional databases (NCBI and 1000 Genomes databases) (Fig 2). However, one SNP, rs149605777 (Fig 2), corresponding to a c.626G>A heterozygous coding variant, was found only in the HIV+HCV- case population (G>A n = 1). This variant leads to a missense Arg209Gln (R209Q) change in the C-terminal cytosolic tail of CLDN6, with a non-attributed MAF (minor allele frequency) in the NCBI database. Prediction tools (PolyPhen 2 and SIFT) showed that this mutation is possibly damaging or tolerated (scores of 0.533 with PolyPhen and 0.43 with SIFT). Human Splicing Finder (HSF) software showed that this c.626G>A substitution potentially creates a splice acceptor site. However, sequencing of cDNAs derived from the patient’s blood cell mRNA showed no evidence of splicing *in vivo* (see Materials and Methods).

In the CLDN9 gene, we identified SNPs already described in databases (Fig 2). There was no statistically significant difference between the control and case populations.

In the OCLN gene, we identified several SNPs and also one rare heterozygous variant, exclusively in the case population (Fig 2). This rare rs147125035 variant was present in only one HIV+HCV- patient and corresponded to a missense c.70C>G substitution with a MAF frequency of 0.006. It leads to a Pro24Ala (P24A) change (Fig 2) in the N-terminus of OCLN. This variant is predicted to be potentially damaging or deleterious (scores of 1 with PolyPhen and 0.12 with SIFT). Interestingly, this rare variant was found in the case patient harboring the CLDN6 mutation described above. HSF software identified no splice site for this variant. Both mutated residues in CLDN6 and OCLN are highly conserved in different species.

We also identified two variants of the SCARB1 gene, both in another case patient (Fig 2): rs5891 (MAF: 0.007) and rs372663606 (no attributed MAF in NCBI), corresponding to heterozygous c.403G>A missense (leading to a p.Val135Ile change) and c.1056G>A synonymous (p.Pro352) substitutions, respectively. HSF software identified no splice site for this variant. The c.403G>A missense substitution is predicted to be benign/tolerated (scores of 0.322 with PolyPhen and 0.5 with SIFT).

Thus, we found that 2 of the 22 HIV+HCV- IDU case patients displayed specific mutations or variants in HCV cellular receptors. Interestingly, one patient harbored two variants of different cellular factors required for HCV entry. The affected residues are highly conserved in different species and are likely damaging. One mutation has no frequency described in databases, while the other variant is rare and was not found in the control population.

## Discussion

The overall seroprevalence of HCV in HIV-infected patients is around 24% [23], but it can reach 92.8% in HIV-1-infected IDU (2). As observed in the Urban Health Study, patients who acquire HIV-1 through intravenous drug use but remain uninfected by HCV are rare [24]. The latter study involved 25 Caucasian HIV+HCV- IDU patients. Here, we selected individuals with prolonged exposure to HCV (at least five years of IDU) and in whom HIV-1 infection was diagnosed before 1995, in order to avoid false resistance to HCV infection. We therefore considered that the HCV-negative patients were very likely resistant, as most or all of them would have been exposed to HCV for several years.

To explore the possibility that variations in HCV cell entry factors might be involved in resistance to HCV infection, we sequenced the genes of four entry factors in which point mutations are known to abolish HCV entry *in vitro* [19–22]. We found variants in CLDN6, OCLN and SCARB1, solely in the cases (2/22, 18%). The corresponding mutations affected residues that are highly conserved in various species and are thus likely to be damaging. Two mutations had no reported database frequency, while the other two variants were rare and not found in the control population. Interestingly, the same case patient harbored one heterozygous mutation in CLDN6, together with one rare heterozygous mutation in OCLN, both of which were likely damaging and involved residues highly conserved in different species. The probability of finding both variants in the same case patient was extremely low. Another case patient harbored two mutations in SCARB1, but prediction tools suggested that they were unlikely to have functional consequences. Once again, the likelihood of the two variants occurring in the same patient was extremely low. The combinations of new mutations or rare variants specifically present in HIV-infected but HCV-uninfected patients but not in HIV and HCV-infected patients suggest that HCV resistance might be a multigenic phenomenon.

While many studies have explored the role of genetic variants of chemokine receptors in the susceptibility and progression of HIV disease, the full coding regions of several coreceptor genes have rarely been sequenced in the same sexually exposed but HIV-uninfected population [25–36]. In keeping with our results, a large proportion of individuals who remain HIV-uninfected despite repeated sexual exposure harbor no mutations in HIV entry coreceptors; only 10–18% of these individuals have heterozygous mutations and as few as 1–2.8% have homozygous mutations in CCR5 (mostly CCR5Δ32) [25, 26, 37–41]. This suggests that host resistance to HIV may involve other mechanisms besides coreceptor mutations, especially as heterozygous CCR5Δ32 mutations simply slow HIV disease progression, without preventing infection [25, 26, 42]. The full coding regions of several coreceptor genes have never previously been sequenced in an HIV-resistant IDU population [25, 26, 28, 29, 32, 40, 43, 44]. Only known specific variants of CCR5, CCR2, SD F1 RANTES have so far been studied. The only study of HCV-infected, HIV-uninfected patients (HCV infection being a marker of intravenous exposure to HIV) revealed a strong protective effect of a high copy number of the *CCL3L1* gene, which encodes a CCR5 ligand [25, 45].

In conclusion, in the first study of its type, we detected mutations in HCV entry factors in two (18%) of 22 HIV-infected patients who were highly exposed to HCV but remained uninfected. However, most such patients had no mutations in coreceptors in which single mutations are known to abolish HCV entry *in vitro*, highlighting the complexity of the mechanisms governing HCV resistance and entry *in vivo*. The observed combination of a new mutation with specific variants in two HCV-exposed but uninfected patients suggests that resistance to HCV may be associated with other, additive genetic factors, as also suggested for resistance to HIV. Whole-genome studies are now needed to identify the different cellular factors involved in HCV resistance.
